# Transporters and Efflux Pumps Are the Main Mechanisms Involved in *Staphylococcus epidermidis* Adaptation and Tolerance to Didecyldimethylammonium Chloride

**DOI:** 10.3390/microorganisms8030344

**Published:** 2020-02-29

**Authors:** Urška Ribič, Jernej Jakše, Nataša Toplak, Simon Koren, Minka Kovač, Anja Klančnik, Barbara Jeršek

**Affiliations:** 1 Department of Food Science and Technology, Biotechnical Faculty, University of Ljubljana, Jamnikarjeva 101, SI-1000 Ljubljana, Slovenia; ursa.ribic@gmail.com (U.R.); anja.klancnik@bf.uni-lj.si (A.K.); 2 Department of Agronomy, Biotechnical Faculty, University of Ljubljana, Jamnikarjeva 101, SI-1000 Ljubljana, Slovenia; jernej.jakse@bf.uni-lj.si; 3 Omega d.o.o., Dolinškova 8, SI-1000 Ljubljana, Slovenia; natasa.toplak@omega.si (N.T.); simon.koren@omega.si (S.K.); minka.kovac@omega.si (M.K.)

**Keywords:** *Staphylococcus epidermidis*, RNA-seq, didecyldimethylammonium chloride, adaptation mechanisms, tolerance mechanisms

## Abstract

*Staphylococcus epidermidis* cleanroom strains are often exposed to sub-inhibitory concentrations of disinfectants, including didecyldimethylammonium chloride (DDAC). Consequently, they can adapt or even become tolerant to them. RNA-sequencing was used to investigate adaptation and tolerance mechanisms of *S. epidermidis* cleanroom strains (SE11, SE18), with *S. epidermidis* SE11Ad adapted and *S. epidermidis* SE18To tolerant to DDAC. Adaptation to DDAC was identified with up-regulation of genes mainly involved in transport (thioredoxin reductase [*pstS*], the arsenic efflux pump [gene ID, SE0334], sugar phosphate antiporter [*uhpT*]), while down-regulation was seen for the Agr system (*agrA*, *arC*, *agrD*, *psm*, SE1543), for enhanced biofilm formation. Tolerance to DDAC revealed the up-regulation of genes associated with transporters (L-cysteine transport [*tcyB*]; uracil permease [SE0875]; multidrug transporter [*lmrP*]; arsenic efflux pump [*arsB*]); the down-regulation of genes involved in amino-acid biosynthesis (lysine [*dapE*]; histidine [*hisA*]; methionine [*metC*]), and an enzyme involved in peptidoglycan, and therefore cell wall modifications (alanine racemase [SE1079]). We show for the first time the differentially expressed genes in DDAC-adapted and DDAC-tolerant *S. epidermidis* strains, which highlight the complexity of the responses through the involvement of different mechanisms.

## 1. Introduction

*Staphylococcus epidermidis* is a member of the coagulase-negative *Staphylococcus* group, and it is the most common bacterial species of human skin and mucosa [1,2,3]. *S. epidermidis* is a leading cause of infections related to medical devices, and as highlighted by the European Centre for Disease Control, the resistance to multiple antimicrobial agents among *S. epidermidis* strains is a concerning trend that might limit treatment options for infections that are already difficult to treat [4]. A reduction in *S. epidermidis* prevalence would eliminate the reservoir of resistance genes and decrease the risk of further transfer of resistance genes and resistance-associated mobile genetic elements [5]. *S. epidermidis* strains are also known to easily adapt to different environmental changes. Thus, the presence of low concentrations of disinfectants will provide the opportunity for these bacteria to adapt and/or to develop tolerance [6,7].

*S. epidermidis* strains are one of the most common cleanroom isolates. Cleanrooms are places that are considered to be microbially reduced environments [8] with defined and controlled air quality (e.g., generally using high-efficiency particulate air-filtration systems), particles in the air, temperature and humidity. Cleanrooms ensure the quality and safety of many production areas, where contamination with microorganisms and particles is critical, such as in surgical units, manufacture of pharmaceuticals, food production and biosafety laboratories [8,9]. Therefore, disinfection of cleanrooms is a common procedure that is very important for maintenance of low levels of microbial burden. 

Quaternary ammonium compounds (QACs) and didecyldimethylammonium chloride (DDAC) are commonly used as disinfectants, as they have broad-spectrum activities [10,11,12,13]. However, regular surface disinfection can result in low levels of QACs maintained in the environment. Therefore, bacteria might adapt towards an individual antimicrobial it is exposed to, or even develop tolerance to the disinfectants used [14]. It is also known that adaptation or tolerance to QACs can lead to cross-tolerance to other antimicrobial substances, such as other biocides and antibiotics [15].

Adaptation is usually a reversible biological property, such that it is lost with the removal of the disinfectant. However, bacterial tolerance to a disinfectant is a permanent change that allows tolerant cells to grow at higher concentrations of the disinfectant than non-tolerant cells. Here, tolerance remains even after the removal of the disinfectant, and it can be transferred across generations [16]. Several bacterial adaptation and tolerance mechanisms are known to be involved, such as modulation of cell growth rate, biofilm formation, efflux pump activity, fatty-acid profile, and the cell size, cell membranes and cell wall [16,17,18,19]. However, in general, tolerance development to QACs is still not fully understood [13].

RNA-sequencing (RNA-seq) is a common tool for analysis of differentially expressed genes (DEGs) [20] and we used this method to identify candidate genes and the underlying *S. epidermidis* biological pathways involved in adaptation and development of tolerance to DDAC. The transcriptomes of staphylococci (*S. epidermidis*, *S. aureus*) have been analyzed after exposure to environmental stresses [7,21,22], but none of these studies have been directly associated to cleanroom strains and adaptation or tolerance to DDAC. Adaptation of the OJ82 seafood strain of *Staphylococcus* spp. to high salt concentrations resulted in higher expression of genes involved in basic cellular processes, such as translation (ribosomal proteins, tRNA synthetase, translation initiation factor genes) and ATP production, together with membrane synthesis, transport systems, and metabolic pathways [22]. Similar responses in nitrogen metabolic pathways in *S. aureus* and *S. epidermidis* human strains were observed as a response to sub-inhibitory concentrations of sapienic acid [21]. Investigation of the food-strain transcriptome of *S. aureus* grown in the presence of disinfectants (ethanol, chloramine T, their combination) revealed significant up-regulation of genes associated with biofilm formation, transport systems, metabolism pathways and membrane composition [7]. To our knowledge, the precise mechanisms involved in the adaptation and tolerance of cleanroom *S. epidermidis* strains to DDAC has not been studied. 

In the present study, we used the adapted and tolerant *S. epidermidis* cleanroom strains that we had previously obtained by their exposure to sub-inhibitory concentrations of DDAC [19]. We aimed to gain insight into the transcriptomic changes for the biological processes involved in the adaptation and tolerance mechanisms of these *S. epidermidis* cleanroom strains. Using RNA-seq, we analyzed the differential expression of genes through a comparison of the transcriptomes of these adapted and tolerant strains (SE11Ad, SE18To) with the transcriptomes of their original strains before adaptation to DDAC as control strains (SE11, SE18, respectively). To the best of our knowledge, this is the first time that the adaptation and tolerance response mechanisms of *S. epidermidis* strains have been investigated using RNA-seq to study adaptation and tolerance to DDAC, as one of the commonly used QAC disinfectants. 

## 2. Materials and Methods 

### 2.1. Staphylococcus epidermidis Strains and Growth Conditions

The *S. epidermidis* SE11 and *S. epidermidis* SE18 cleanroom strains were used as the control strains (henceforth referred to as SE11, SE18). These strains had been exposed to DDAC (Merck, Darmstadt, Germany) in our previous study [19]. The DDAC-adapted SE11Ad and DDAC-tolerant SE18To *S. epidermidis* strains obtained were used in the present study (henceforth referred to as SE11Ad, SE18To). The DDAC minimal inhibitory concentration (MIC) against SE11Ad was 18.0 µg/mL, while for the control SE11 this was lower, at 4.5 µg/mL. The DDAC MIC against SE18To was 9.0 µg/mL, and against SE18 it was 0.6 µg/mL [19]. 

For RNA isolation, SE11 and SE18 (1 mL) were grown in Tryptic Soya Broth (TSB; Oxoid, Hampshire, UK) (9 mL), while SE11Ad and SE18To were grown in TSB with 18.0 µg/mL and 9.0 µg/mL DDAC, respectively. All of these *S. epidermidis* strains were incubated at 37 °C for 4.5 h with shaking (120 rpm), to a concentration of approximately 10^9^ cells/mL.

### 2.2. Next-Generation Sequencing

#### 2.2.1. RNA Isolation and Quantification

The total RNA of SE11, SE11Ad, SE18 and SE18To were isolated using PureLink RNA mini kit (Thermo Fisher Scientific, Carlsbad, CA, USA), following the manufacturer protocol, with some minor modifications. Cell lysis was performed in lysis solution with 1% 2-mercaptoethanol (98%; Sigma-Aldrich Chemie, Taufkirchen, Germany), and the cells were disrupted using a laboratory mill (Retsch MM400; Retsch GmBH, Haan, Germany) at the maximum speed (1800 rpm) for 35 s, followed by cooling on ice (2 min). During the extraction step, *DNAse* treatment was performed using PureLink DNAse sets (Thermo Fisher Scientific). The total RNA yield and quality were investigated with Qubit RNA HS assay kits (Thermo Fisher Scientific), according to manufacturer protocol, using a fluorometer (Qubit v4; Thermo Fisher Scientific). The messenger RNA (mRNA) fraction from the total RNA was enriched using beads (NEXTflex PolyA; Perkin Elmer, Waltham, MA, USA), according to the manufacturer protocol.

#### 2.2.2. Ion Torrent Library Preparation and Sequencing

RNA-sequencing next-generation sequencing libraries were prepared using Ion Total RNA-seq kits v2 (Thermo Fisher Scientific), following the manufacturer instructions and including four different barcodes. The amounts and size distributions of the prepared library fragments were determined (Labchip GX; Perkin Elmer). Strand-specific sequencing was performed using the Ion Proton system (Thermo Fisher Scientific). Emulsion PCR and the enrichment steps for the four pooled libraries were carried out using Ion P Hi-Q OT2 200 kits (Thermo Fisher Scientific), as described by the manufacturer. Library templates were sequenced with Ion P Hi-Q Sequencing 200 kits (Thermo Fisher Scientific) using a single Ion PI Chip kit v3 (Thermo Fisher Scientific). Signal processing, base calling, and adapter trimming were performed with the Torrent Suite software version 5.10.1 (Thermo Fisher Scientific). The sequences of the raw data have been deposited with the NCBI Sequence Read Archive (SRA) under BioProject SRA accession number PRJNA596062 (SE11, SRR10715330; SE11Ad, SRR10715329; SE18, SRR10715328; SE18To, SRR10715327).

### 2.3. Data Analysis

The bioinformatics analysis was performed using the CLC Genomics Workbench (version 12.0.3) and the CLC Genomics Server (version 11.0.2) tools. The basic quality control of the reads was performed using the ‘QC for Sequencing Reads’ tool (Qiagen, Redwood City, CA, USA). The reference genome used for the mapping sequences was the *S. epidermidis* ATCC12228 genome from NCBI (NC_004461), including its six plasmids (NC_005008, NC_005007, NC_005006, NC_005005, NC_005004, NC_005003) with the gene coordinates. For screening the *qac* genes, the *S. epidermidis* pHOB1 plasmid reference sequence was used (NZ_CP018843.1). The mapping of the sequencing reads to the reference sequences was done with the ‘RNA-seq analysis’ tool using the default settings with the strand-specific option set as ‘Forward’. The corresponding mapping profiles were used in a statistical differential expression test implemented in the ‘Differential expression for RNA-seq tool’, which uses multi-factorial statistics based on a negative binomial generalized linear model. The DEGs were chosen based on fold change (FC) >1 and *p* <0.05. SE11Ad was analyzed in comparison to its control, SE11, and SE18To to its control, SE18. The gene ontologies for biological processes, cellular components and molecular functions for these genes were searched in the UniProt database (https://www.uniprot.org/), based on gene model accession numbers and according to their functional annotations using the Blast2GO command line (version 1.3.0). These DEGs were further analyzed according to the Kyoto Encyclopaedia of Genes and Genome (KEGG) pathways, in the KEGG mapper tool (https://www.genome.jp/kegg/mapper.html).

## 3. Results and Discussion

Transcriptomic analyses were performed for SE11Ad (DDAC-adapted *S. epidermidis* SE11 strain) and SE18To (DDAC-tolerant *S. epidermidis* SE18 strain). Strand-specific RNA-seq was analyzed using the Ion Torrent technology. Altogether, after default Torrent Suite quality filtering and trimming, 42.26 M reads were obtained for the four transcriptomes studied (Table 1). The majority of the reads mapped to the reference *S. epidermidis* ATCC12228 genome with six plasmids and the *S. epidermidis* pHOB1 plasmid (Table 1). The data are available in the SRA under BioProject accession number PRJNA596062.

The mechanisms of *S. epidermidis* that are involved in adaptation and tolerance to DDAC were studied for the first time according to the DEGs and their involvement in biological pathways and processes. SE11Ad showed 720 DEGs, with 385 up-regulated and 335 down-regulated. SE18To showed 1013 DEGs, with 519 up-regulated and 494 down-regulated (Supplementary Table S1). 

### 3.1. Response Mechanisms to DDAC of the DDAC-adapted S. epidermidis Strain, SE11Ad 

**Figure 1:**
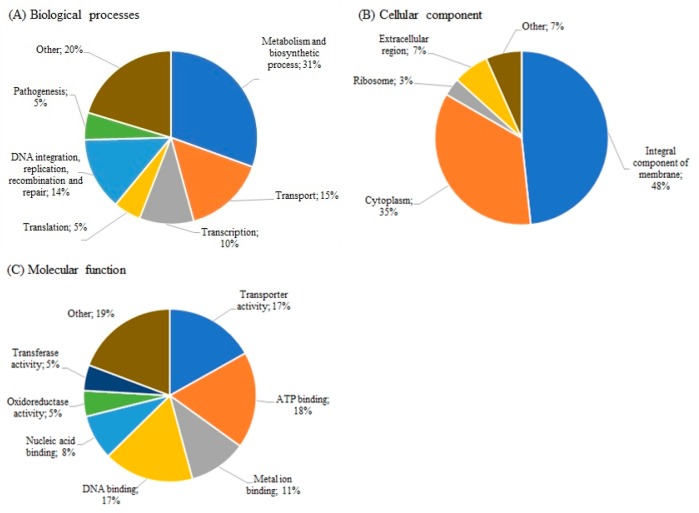
Gene ontology analysis of the significant differentially expressed genes (FC >5, *p* <0.05) of SE11Ad (DDAC-adapted *S. epidermidis* strain) for ‘Biological processes’ **(A)**, ‘Cellular component’ **(B)** and ‘Molecular function’ **(C)**.

Detailed analysis of SE11Ad revealed 111 significant DEGs when analyzed in comparison to its control SE11. Among these, 37 genes were up-regulated and 74 genes were down-regulated (Supplementary Table S2). According to gene ontology analysis, different metabolic and biosynthetic processes, transport systems, and DNA transformations were the most significant biological processes (Figure 1A). ‘Integral component of membrane’ was the main cellular component (Figure 1B), and ‘ATP-binding’, ‘Transporter activity’, ‘DNA-binding’ and ‘Metal-ion binding’ were the main molecular function activities (Figure 1C) that characterized these DEGs in SE11Ad.

The KEGG pathway analysis defined the most representative cellular pathways of these DEGs involved in SE11Ad adaptation to DDAC (Supplementary Table S2). The highest number of DEGs, along with their higher fold changes, were identified among the metabolic and biosynthetic pathways. Glycerol ester hydrolase is involved in lipid metabolism, and showed the highest fold change (*gehD* [gene ID, SE0185], -495.5-fold). Due to its lipase activity, it was defined as an important virulence factor that enables *S. epidermidis* to grow on a lipid-rich skin environment [23,24]. This gene was also down-regulated in an *S. epidermidis* RNAIII mutant with a reduced quorum sensing (Agr) system, which suggested that *gehD* is Agr-dependent [25]. Seventeen other genes were also involved in different metabolic pathways and in biosynthesis of secondary metabolites and antibiotics (Table 2). Three genes involved in the shikimic acid pathway were down-regulated (chorismate mutase [SE1599], -26.1-fold; shikimate kinase [SE1224], -6.2-fold; 3-phosphoshikimate 1-carboxyvinyltransferase [SE1153], -5.8-fold). This metabolic pathway leads to the biosynthesis of chorismic acid, a precursor of aromatic amino acids, folate, ubiquinone, and other isoprenoid quinones [26]. Furthermore, dihydrolipoamide dehydrogenase was down-regulated (SE1199, -11.1-fold), similar to that seen as the manuka honey effect in *S. aureus* [27]. Reduced expression of these metabolism-associated enzymes might be related to the SE11Ad adaptation response as decreased energy consumption of the cells for metabolism. 

The down-regulated fused signal recognition particle receptor (*ftsY* [SE0910]) is part of the *S. epidermidis* Sec secretion system, and is involved in cell division, as a highly energy demanding process. The expression of *ftsY* was also reduced when *S. epidermidis* cells were exposed to α-mangostin, a natural xanthone with antimicrobial activity [28]. This indicates another energy saving process as an adaptation response.

Another adaptation response identified here for SE11Ad was reduced production of secondary metabolites, as in this way cells can save energy to ‘streamline’ their primary metabolism [29,30]. The down-regulated *Ipk* gene (SE2288, -21.7-fold) is involved in terpenoid biosynthesis, which is essential for cell survival. Terpenoid biosynthesis enzymes have already been investigated as potential drug targets in staphylococci, and decreased expression of this biosynthesis pathway is known to lead to down-regulation of primary metabolism and a shift in the regulation of virulence factors and cell-wall biosynthesis genes [31]. The down-regulated uroporphyrinogen III synthase (SE1344, -5.9-fold) is involved in hem biosynthesis [32], together with cytochrome c oxidase assembly protein subunit 15, hem synthase (SE0814, -8.5-fold) and *hemC* (SE1345, -12.8-fold). Hem biosynthesis reduction has already been shown for biofilm and persister cells (small colony variants) of *S. aureus* [33]. *MiaA* was also down-regulated (SE0981, -8.9-fold), and it encodes tRNA dimethyl allyl transferase, which is responsible for tRNA biosynthesis. Strong repression of genes responsible for DNA synthesis and its metabolism was shown in *S. aureus* exposed to the antimicrobial mupirocin, along with the same for those for translation processes [34]. Similar effects were reported in *S. aureus* exposed to ethanol, where genes for transcription, translation and nucleotide biosynthesis were generally down-regulated [35]. It has been suggested that exposure to this antimicrobial agent triggers a global adaptation response that is intended to adjust the cell biosynthetic pathways to the availability of energy and the required precursors. 

Genes involved in quorum sensing were highly down-regulated (Table 2). Among these, four were from the accessory gene regulator (Agr) system (*agrA* [SE1638], -81.3-fold; *agrC* [SE1637], -77.0-fold; *agrD* [SE1636], -391.5-fold) and phenol soluble modulin β1/β2 (SE0484, -285.1-fold). The Agr system is considered as the main quorum-sensing mechanism in Gram-positive bacteria, including *S. epidermidis* strains [36]. The reduced expression of these genes might be directly associated with the adaptation of SE11Ad to DDAC, as down-regulation of the Agr system is associated with increased biofilm formation and decreased formation of phenol-soluble modulins (low-molecular-weight toxins) and other exoenzymes [25,36]. Through the Agr system, cells maintain energy for the vital activities connected to biofilm formation, which can protect them against DDAC. This regulation is usually described as initial bacterial colonization and development of chronic human infections [25,36]. However, for SE11Ad, the reduced expression of genes involved in the Agr system was identified as the mechanism that might have a large contribution in adaptation to DDAC, as reduced expression of the genes of the Agr system enhance biofilm formation as a response to DDAC. Indeed, stronger biofilm formation was detected in SE11Re, in comparison to the control SE11 [19]. The Agr system also regulates some extracellular proteases, such as V8 protease (SE1543, -534.6-fold). This protease is involved in extracellular protein cleavage and has been associated with protein-based biofilm removal [37,38]. As reported previously, in *S. epidermidis*, a reduced Agr system is connected with reduced production of extracellular enzymes, like proteases [25]. Down-regulation of V8 protease appears to contribute to greater biofilm formation, as an adaptation response to DDAC.

Other highly down-regulated genes included putative transposases for insertion-sequence-like elements (SE0079, -1795.91-fold; SE0090, -636.85-fold; *BUM85_RS13075*, -1447.36-fold) (Supplementary Table S2). Transposases are normally responsible for genetic recombination and the repair of genetic material, along with another down-regulated gene that encodes the recombination protein recR (*recR*, -13.86-fold). *S. epidermidis* has many mobile genetic elements that enable mobilisation and horizontal gene transfer. Insertion sequences are usually small DNA sequences that include one or two transposase genes [39]. Often, these are associated with resistance genes, which can randomly move to new genomic locations. This is one of the most concerning phenomena of the spread of resistance genes among bacterial cells of the same, or even different, species, and this genome plasticity provides bacteria with rapid adaptation to changing environmental conditions [39,40,41]. This mechanism is known to occur in stressful environments [42], and it might increase the adaptation of SE11Ad to DDAC. As high repression of putative transposase for insertion-sequence-like elements was seen for SE11Ad, they appear to be involved in adaptation. 

There were eight highly up-regulated genes for SE11Ad among the transporters, which are responsible for the transport of different substances, such as mineral and organic ions, oligosaccharides, lipids, sterols, peptides, proteins, metals, amino acids and drugs and other antimicrobials (Table 2). Transporters have been suggested to be defined as virulence factors, as they are responsible for the transport of numerous substrates that are essential for virulence and survival in stress environments [43]. Thioredoxin reductase (*pstS* [SE1070]) was the most up-regulated gene for SE11Ad, with the highest fold-change (124.9-fold). It is part of a transporter involved in the thiol-specific redox system. This system is responsible for maintenance of cell homeostasis and protection of the cell from oxidising compounds, and therefore it is involved in cell defense against toxic compounds [44]. A phosphate transporter gene (*pstC* [SE1069]) was also highly up-regulated (38.5-fold). Together with PstA, this PstC protein forms a channel for the transport of phosphate across the cytoplasmic membrane [45]. Additionally, *pstB* (SE1067) was up-regulated (8.00-fold). Usually, this phosphate transport is more expressed in environments with limited phosphate. When *S. aureus* was exposed to the antimicrobial peptide ranalexin, similar effects were observed [46], and this was thus suggested as a *Staphylococcus* adaptation mechanism. Among the ABC transporters, genes for amino-acid transport were also up-regulated, such as the D-methionine transport system permease (*metI* [SE2321]), the methionine transport ATP-binding protein (*metN* [SE2322]), and the L-cystine transport system permease (*tcyB* [SE1992]). A supply of these particular amino acids is needed for growth and survival of SE11Ad [47]. The oligopeptide transport system permease (*oppC* [SE0681]) was also up-regulated, which is associated with the transport of small peptides for cell-wall biosynthesis [48]. This is part of the *opp* operon, which is necessary for survival for *S. aureus* [49].

As well as these transporters, the efflux systems were involved in the response of SE11Ad to this DDAC adaptation (Table 2). The up-regulation of genes involved in different efflux systems have also been reported previously after exposure and adaptation to antimicrobials, such as for *S. aureus* after exposure to silver nanoparticles [50], for *Pseudomonas aeruginosa* adapted to benzalkonium chloride [51], and for *S. epidermidis* adapted to ethidium bromide [52]. An important efflux system for the arsenic efflux pump (SE0334) showed an increase in 11.8-fold (Table 2). This efflux pump actively exports toxic arsenic compounds from cells [53]. The arsenic efflux system has multidrug-resistance members [54], and according to these data, we suggest that this system contributes to the active export of DDAC in SE11Ad, although further supporting evidence is needed. Among the two component systems, the *uhpT* gene that encodes a sugar-phosphate antiporter was highly up-regulated (11.5-fold). This is involved in phosphomycin resistance in methicillin-resistant *S. aureus* strains [55].

According to the metabolic pathways, the most up-regulated gene in metabolism encodes carbamate kinase (*arc* [SE0228], 20.7-fold). This is involved in arginine metabolism through the arginine deiminase pathway and is known as an important pathogenesis mechanism as its final product is ammonia, which increases cytoplasmic pH and therefore protects cells from toxic acidic conditions and maintains cytosolic pH homeostasis [56].

### 3.2. Response Mechanisms to DDAC of the DDAC-tolerant S. epidermidis Strain, SE18To 

**Figure 2:**
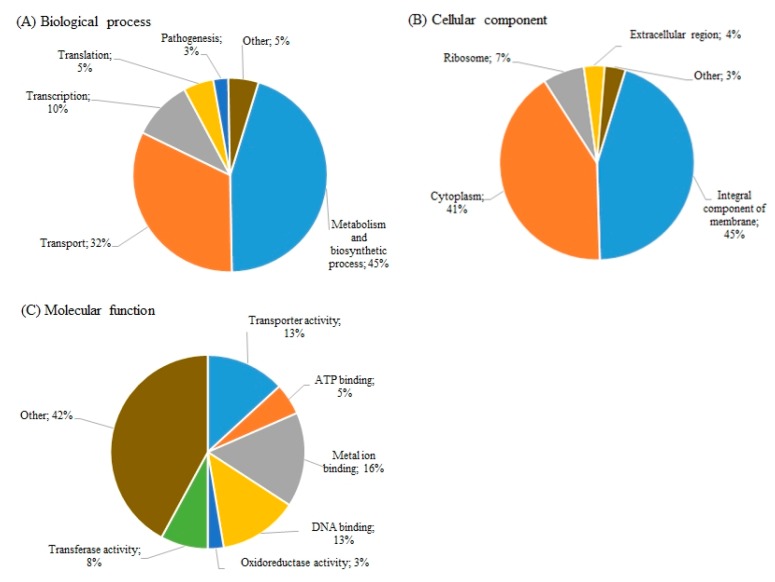
Gene ontology analysis of the significant differentially expressed genes (FC >5, *p* <0.05) of SE18To (DDAC-tolerant *S. epidermidis* strain) for ‘Biological processes’ (**A**), ‘Cellular component’ (**B**) and ‘Molecular function’ (**C**).

Detailed analysis of SE18To identified 42 DEGs (FC >5, *p* <0.05), among which 21 were up-regulated and 21 were down-regulated (Supplementary Table S2). Gene ontology analysis revealed different metabolism and biosynthetic processes and transport systems as the most significant biological processes associated with these DEGs (Figure 2A). According to the cellular component, the majority of the DEG products were ‘Integral components of membrane’ (Figure 2B), and ‘Metal-ion binding’, ‘Transporter activity’ and ‘DNA-binding’ were the main molecular functions that characterized these DEGs in SE18To (Figure 2C).

According to KEGG analysis, the most down-regulated genes were identified as among those involved in amino-acid biosynthesis. The *dapE* gene (SE2220, 3-4.0-fold) encodes succinyl-diaminopimelate desuccinylase (Table 3). This enzyme is involved in the biosynthesis of lysine, an important amino acid in bacterial cell-wall synthesis [57]. SE1079 was highly down-regulated (-16.9-fold) and encodes alanine racemase, which is involved in cell-wall synthesis and growth in *S. aureus* [58]. It converts L-alanine to D-alanine, which is a precursor of the pentapeptide moiety that links the glycan chains in peptidoglycan. A reduction in these peptidoglycan linkages results in greater elasticity of the *S. aureus* cell wall [59]. Alanine racemase has also been related to enterococci resistance against vancomycin, an antibiotic that targets the cell wall [18]. Modification of the peptidoglycan and bacterial cell wall are highly regulated mechanisms, and these can occur as responses to different environmental stimuli, with the basic aim being to protect the inner content of cells [16,48]. Such cell-membrane and cell-wall modifications usually result in thickening of the staphylococcal cell wall as a resistance mechanism, to help protect the cell against antimicrobials [18]. Other genes involved in amino-acid biosynthetic pathways were down-regulated, including (SE0275, -7-fold); this gene encodes 1-(5-phosphoribosyl)-5-[(5-phosphoribosylamino) methylideneamino] imidazole-4-carboxamide isomerase, which is involved in L-histidine biosynthesis. Down-regulation of *hisA* was also seen for *Staphylococcus xylosus* after exposure to aspirin [60]. Ketol-acid reductoisomerase (SE1657, -8.1-fold) is involved in branched-chain amino-acid biosynthesis [61]. Cystathionine β-lyase (*metC* [SE2380], -7.8-fold) is an enzyme that primarily converts L-cystathionine to homocysteine, pyruvate and ammonia, and it is involved in methionine biosynthesis [62]. A reduction in the basic metabolic processes and growth of cells is a survival strategy [63]. This down-regulation of the biosynthesis of amino acids and secondary metabolites indicates major changes in the amino-acid profile. Modifications to amino acids have been shown to be a response mechanism for a changing environment. This was shown by an analysis of the amino-acid profile of *S. aureus* after exposure to hydrogen peroxide [64]. Modifications to the amino-acid profile influence many biological pathways in cells, as amino acids are required for protein synthesis, and used for synthesis of the cell-wall and nucleic acids, and they can be used as energy substrates [64]. Reductions in cytoplasmic amino acids has been associated with slower rates of metabolism, to conserve energy for basal metabolism [65]. Modifications to cell membranes and the cell wall are known to be important to maintain cellular homeostasis, by the protection of cells from changes in the environment. This is achieved by maintenance of cell shape and turgor pressure, and is involved in cell division, energy production and permeability regulation [18].

The most up−regulated gene for SE18To was SE0875, which encodes uracil permease (27.6−fold), although this does not have a defined KEGG status (Supplementary Table S2). This is an electrochemically driven transporter that is responsible for transport of the nucleotide uracil. In a vancomycin−resistant *S. aureus* strain, this gene increased purine biosynthesis [66]. A shift in purine metabolism is one of the most common resistance responses in methicillin−resistant *S. aureus* [67]. Overall, up−regulation of transport, and particularly of the ABC transporters, was also reported in an analysis of the transcriptomes of ciprofloxacin−resistant *S. aureus* strains [68]. The transporter gene for L−cysteine, *tcyB*, was among the most up−regulated transporters in SE18To (SE1992, 16.0−fold). Together, *tcyA*, *tcyB* and *tcyC* encode the ABC transporter, which is responsible for uptake of cysteine, as required for cell growth. It has been suggested that in an environment with limited cysteine, the genes for this cysteine transporter are up−regulated [69]. As the biosynthesis of amino acids in SE18To was reduced, import from the environment provides the source for the cell needs. Other transport systems were also up−regulated, including the zinc transport system permease (*znuB* [SE1242], 6.8−fold), a part of the zinc transporter that is responsible for transporting zinc and other metals into and out of the cell, and therefore for the regulation of cell metal homeostasis. The up−regulated *lmrP* gene (SE0242, 7.0−fold; Supplementary Table S2) encodes a transporter protein that is an orthologue of the *Lactococcus lactis* LmrP multidrug efflux protein. This actively exports cationic amphiphilic substrates out of the cell, and is predicted to also export drugs in *S. epidermidis* [70,71]. Another multidrug efflux pump that was up−regulated was the arsenic efflux pump (a*rsB* [SE0135], 5.97−fold). This energy−dependent efflux system belongs to the multi−drug−resistance protein family, which is involved in the active export of toxic compounds from cells [53,54].

The best described mechanism of bacterial tolerance to QACs involves the overexpression of efflux systems [16,72,73,74]. It is known that *S. epidermidis* has nearly 30 different drug efflux pumps, some of which are predicted to actively export QACs out of the cell [75]. The nature of DDAC and its non−specific membrane activity mean that it can enter cells by interacting with the phospholipid bilayer, which disrupts the membrane diffusive forces and ion gradients. This damage often leads to membrane lysis and cell death [13,76]. Active export of DDAC out of cells is a common mechanism of tolerance that eliminates the toxic DDAC from cells, to prevent cell damage [13]. This might be the major tolerance mechanism in SE18To.

### 3.3. L−cystine Transport and Arsenic Efflux Systems in DDAC−adapted and −tolerant S. epidermidis Strains 

Results of the detailed analysis of SE11Ad and SE18To revealed that L−cystine transport and arsenic efflux are common responses to increased DDAC concentrations. The gene that encodes L−cystine permease (*tcyB* [SE1992]), which is part of the L−cystine amino−acid transport, was overexpressed in both SE11Ad and SE18To. In SE11Ad, *tcyB* was up−regulated 10.5−fold, and in SE18To, 16.0−fold. Up to 12 different amino acids are required for the growth of staphylococci, which include cysteine [77]. L−cysteine is also part of the low−molecular−weight thiols that have protective role in cells when they are exposed to reactive oxygen species, reactive electrophilic species, antibiotics and heavy metals [78]. Exposure of *S. aureus* to diamide leads to S−thiolation, and formation of disulphide bonds among proteins and low−molecular−weight thiols, and these products protect cytoplasmic proteins from irreversible oxidation [79]. Along with diamide, cysteine metabolism is connected to hydrogen peroxide, superoxide, nitric oxide, thiol−reactive electrophiles and metal ions [80]. We suggest that up−regulation of the *tcyB* gene that is involved in the uptake of L−cysteine is an important adaptation and tolerance mechanism for maintenance of cellular homeostasis in both SE11Ad and SE18To. These cells have greater need for L−cysteine to form low−molecular−weight thiols, and uptake of L−cysteine from the environment is preferred over de−novo synthesis [18,81].

The gene that encodes the arsenic efflux system (SE0334 for SE11Ad; *arsB* [SE0135] for SE18To) was also up−regulated in both of these strains. In SE11Ad, SE0334 was up−regulated 11.8−fold, and in SE18To, *arsB* was up−regulated 6.0−fold (Supplementary Table S2). There are many different arsenic efflux systems in cell membranes, and the *ars* operon is the most studied and known of these, although other novel arsenic efflux pumps have also been described [53]. Usually, these are considered as multidrug−resistance members of efflux systems [54]. These efflux systems represent another common mechanism when these *S. epidermidis* strains are adapted or tolerant to DDAC. 

### 3.4. RNA−sequencing Results and Phenotypic Properties of These S. epidermidis Strains

The RNA−seq results (Table 2 and Table 3) show that genes involved in transport systems and efflux pumps were highly up−regulated in SE11Ad and SE18To. These results confirm our previous study, where we showed increased activity of the efflux pumps in SE11Ad and SE18To, based on an acridine−orange assay [19].

SE11Ad and SE18To showed down−regulation of genes for different metabolic pathways, including the biosynthesis of amino acids and secondary metabolites, whereby the cells will be saving energy for their basic metabolic processes through the reduction in processes that are not crucial for survival. The SE11Ad and SE18To cells showed decreased sizes after the DDAC treatments, in comparison to the control SE11 and SE18 cells, respectively, when examined using scanning electron microscopy [19]. Modifications to the metabolic pathways are related to decreasing cell size, although many different pathways were detected using the RNA−seq analysis here. The reduction in growth and regulation of the basal metabolic activity of cells represent adaptation responses and survival strategies, along with other metabolic changes, to reduce the harmful effects of antimicrobials [63,65,67].

With this RNA−seq analysis, we also identified the significant down−regulation of genes involved in the Agr system, and thus these cells increased biofilm formation, which is a known adaptation mechanism in *S. epidermidis* strains [82]. In our previous study, we showed that at the phenotypic level, SE11Ad increased biofilm formation compared to the control SE11 [19]. Biofilm formation protects cells from the harmful effects of antimicrobials as this limits the diffusion into the cells, and so a shift to biofilm production is one of the adaptation response mechanisms. 

## 4. Conclusions

This analysis of the transcriptomes of these DDAC−adapted (SE11Ad) and DDAC−tolerant (SE18To) *S. epidermidis* strains revealed for the first time the most significant genes, and therefore mechanisms, that are involved in the adaptation and tolerance responses of *S. epidermidis* to this commonly used disinfectant. 

The transcriptomic analysis of SE11Ad showed that adaptation to DDAC comprises different mechanisms, among which the most relevant were up−regulation of the different transporters (*pstS*, *pstC*, *pstB, MetI, MetN*) and efflux pump systems (arsenic efflux pump, *tcyB*), and down−regulation of the Agr system (*AgrA*, *ArC*, *AgrD*, *psm*, V8 protease), the consequence of which was greater biofilm formation. Tolerance to DDAC for SE18To was identified with the down−regulation of genes involved in the metabolic and biosynthetic pathways that are connected to peptidoglycan and cell−wall synthesis, while the most up−regulated genes were for transporters, which suggests that this represents another resistance mechanism. There was increased expression of genes associated with efflux systems (multidrug transporters, LmrP homologue, arsenic efflux pump) as one of the main mechanisms in this *S. epidermidis* tolerance to DDAC. 

The continuing emergence of resistant bacterial strains is a well−known phenomenon, although tolerance to disinfectants is a relatively new aspect. Adaptation and tolerance development to DDAC (as a commonly used disinfectant) in *S. epidermidis* (the most common bacterium associated with cleanrooms) had not been studied to date. Using RNA−seq here, we have revealed that many genes are involved in these complex adaptation and tolerance mechanisms. We have shown that multiple mechanisms have co−developed during adaptation and tolerance to DDAC in these cleanroom *S. epidermidis* strains. These mechanisms of adaptation and tolerance to DDAC that we have identified for cleanroom *S. epidermidis* strains will help in the targeting of *S. epidermidis* strains in terms of the development of disinfectants and disinfection regimes, to control the incidence and spread of bacterial adaptation and tolerance to DDAC. 

## Figures and Tables

**Table 1:** RNA sequencing statistics.

*S. epidermidis*	Condition	Library			
Strain		N°. of Reads	Data Yield (bp)	Average Length (bp)	Mapped to Reference Genome (%)
SE11	Control	6,748,314	796,550,256	118.04	85.46
SE11Ad	Adapted	6,939,698	1,284,519,656	185.01	96.02
SE18	Control	13,150,201	1,826,332,974	138.88	91.12
SE18To	Tolerant	15,425,027	1,945,548,307	126.13	95.63

**Table 2:** The most significant differentially expressed genes (FC >5, *p* <0.05) in SE11Ad, the didecyldimethylammonium chloride (DDAC)-adapted *S. epidermidis* strain, when exposed to the adapted concentration of 18.0 µg/mL DDAC, according to the KEGG pathways.

Gene Expression	KEGG Pathway	Gene ID	Gene	Fold Change	Product
Down-	Transporters	SE0405		−767.0	Lipoprotein; ABC transporter
regulation		SE0407		−250.9	ABC transporter ATP-binding protein
		SE0910		−5.6	Signal recognition particle
	Quorum sensing	SE1543		−534.6	V8 protease
		SE1636	*agrD*	−391.5	AgrD protein
		SE0484	*psm β1/β2*	−285.1	Phenol soluble modulin *β*1/ *β*2
		SE1638	*agrA*	−81.3	Accessory gene regulator A
		SE1637	*agrC*	−77.0	Accessory gene regulator C
		SE0910		−5.6	Signal recognition particle
	Metabolism and	SE0185	*gehD*	−495.5	Glycerol ester hydrolase
	biosynthesis of	SE1599		−26.1	Chorismate mutase
	secondary	SE2288	*ipk*	−21.7	4-Diphosphocytidyl-2C-methyl-D-erythritol kinase
	metabolites	SE0857	*mraY*	−15.1	Phospho-N-acetylmuramoyl-pentapeptide transferase
		SE2081		−14.3	Beta-subunit of L-serine dehydratase
		SE2311	*gltD*	−14.1	Glutamate synthase subunit *β*
		SE1345	*hemC*	−12.8	Porphobilinogen deaminase
		SE0349		−11.9	Phosphomethylpyrimidine kinase
		SE1199		−11.1	Dihydrolipoamide dehydrogenase
		SE0981	*miaA*	−8.9	tRNA d(2)-isopentenylpyrophosphate transferase
		SE0514	*nrdF*	−8.8	Ribonucleotide-diphosphate reductase subunit *β*
		SE1305		−8.7	Iron–sulphur cofactor synthesis protein-like protein
		SE0814		−8.5	Heme synthase
		SE1889	*murQ*	−7.6	N-acetylmuramic acid-6-phosphate etherase
		SE1596	*nadE*	−7.1	NAD synthetase
		SE1224		−6.2	Shikimate kinase
		SE1344		−5.9	Uroporphyrinogen III synthase
		SE1153		−5.8	3-Phosphoshikimate 1-carboxyvinyltransferase
Up-	Transporters and	SE1070	*pstS*	124.9	Thioredoxin reductase
regulation	efflux pump	SE1069	*pstC*	38.5	Phosphate ABC transporter
		SE2321	*metI*	19.6	ABC transporter permease
		SE0334		11.8	Arsenic efflux pump protein
		SE1992	*tcyB*	10.5	ABC transporter permease
		SE0681	*oppC*	8.1	Oligopeptide transport system permease OppC
		SE1067	*pstB*	8.0	Phosphate transporter ATP-binding protein
		SE2322	*metN*	5.4	ABC transporter ATP-binding protein
	Two-component system	SE0164	*uhpT*	11.5	Sugar phosphate antiporter
	Metabolism and	SE0228	*arcC*	20.7	Carbamate kinase
	biosynthesis of	SE2175	*sat*	17.3	Sulphate adenylyltransferase
	secondary	SE2197		13.4	Alkaline phosphatase
	metabolites	SE0319	*ispD*	9.0	2-C-methyl-D-erythritol 4-phosphate cytidylyltransferase
		SE1654		8.0	Dihydroxy-acid dehydratase
		SE2174		7.1	Adenylyl-sulphate kinase
		SE2333		7.0	NADH dehydrogenase subunit 5
		SE2323		6.5	Cystathionine gamma-synthase
	Drug resistance	SE0681	*oppC*	8.1	Oligopeptide transport system permease OppC

**Table 3:** The most significant differentially expressed genes (FC >5, *p* <0.05) in SE18To, the didecyldimethylammonium chloride (DDAC)-tolerant *S. epidermidis* strain, when exposed to the adapted concentration of 9.0 µg/mL DDAC, according to the KEGG pathways.

Gene Expression	KEGG Pathway	Gene ID	Gene	Fold Change	Product
Down−	Metabolism and	SE2220	*dapE*	−34.0	Succinyl−diaminopimelate desuccinylase
regulation	biosynthesis of	SE1079		−16.9	Alanine racemase
	secondary	SE0181		−16.3	8−Amino−7−oxononanoate synthase
	metabolites	SE2600		−8.8	Thiazole synthase
		SE0486		−8.5	6−Pyruvoyl tetrahydrobiopterin synthase
		SE1657		−8.0	Ketol−acid reductoisomerase
		SE2380	*metC*	−7.8	Cystathionine beta−lyase
		SE0275	*hisA*	−7.0	Imidazole−4−carboxamide isomerase1−(5−phosphoribosyl)−5−[(5− phosphoribosylamino)methylideneamino] imidazole−4−carboxamide isomerase
	Transporters	SE2400		−9.9	vraD protein
		SE2255		−7.7	Transport system protein
Up−	Transporters	SE1992	*tcyB*	16.0	ABC transporter permease
regulation		SE2248	*secY−2*	11.8	Accessory Sec system protein translocase subunit SecY2
		SE1242	*znuB*	6.8	ABC transporter
		SE0871	*lspA*	5.2	Lipoprotein signal peptidase
	Metabolism and	SE0274		8.0	Imidazole glycerol phosphate synthase subunit HisH
	biosynthesis of	SE0510		8.0	7−Cyano−7−deazaguanine reductase
	secondary metabolites	SE0873		7.0	Bifunctional pyrimidine regulatory protein PyrR uracil phosphoribosyltransferase
	Ribosome	SE1017	*rpmG2*	5.3	50S ribosomal protein L33 2
		SE0913	*rpsP*	5.6	30S ribosomal protein S16
	Drug resistance	SE1606		5.5	Penicillinase repressor

## Supplementary Materials

The following Supplementary Materials are available online at https://www.mdpi.com/2076-2607/8/3/344/s1. Table S1. Differentially expressed genes (FC >1, *p* <0.05) in SE11Ad and SE18To, the didecyldimethylammonium chloride (DDAC)−adapted and DDAC−tolerant, respectively, *S. epidermidis* strains. Table S2. Analysis of the differentially expressed genes (FC >5, *p* <0.05) in SE11Ad and SE18To, the didecyldimethylammonium chloride (DDAC)−adapted and DDAC−tolerant, respectively, *S. epidermidis* strains, according to the gene ontology and KEGG pathways.
